# Race and ethnic minority, local pollution, and COVID-19 deaths in Texas

**DOI:** 10.1038/s41598-021-04507-x

**Published:** 2022-01-19

**Authors:** Annie Xu, Ted Loch-Temzelides, Chima Adiole, Nathan Botton, Sylvia G. Dee, Caroline A. Masiello, Mitchell Osborn, Mark A. Torres, Daniel S. Cohan

**Affiliations:** 1 Department of Earth, Environmental and Planetary Sciences, Rice University, Houston, USA; 2 Department of Operations Research and Financial Engineering, Princeton University, Princeton, USA; 3 Department of Economics, Rice University, Houston, USA; 4 Department of Civil and Environmental Engineering, Rice University, Houston, USA

**Keywords:** Environmental economics, Diseases, Risk factors

## Abstract

The costs of COVID-19 are extensive, and, like the fallout of most health and environmental crises in the US, there is growing evidence that these costs weigh disproportionately on communities of color. We investigated whether county-level racial composition and fine particulate pollution (PM_2.5_) are indicators for COVID-19 incidence and death rates in the state of Texas. Using county-level data, we ran linear regressions of percent minority as well as historic 2000–2016 PM_2.5_ levels against COVID-19 cases and deaths per capita. We found that a county's percent minority racial composition, defined as the percentage of population that identifies as Black or Hispanic, highly correlates with COVID-19 case and death rates. Using *Value-of-Statistical-Life* calculations, we found that economic costs from COVID-19 deaths fall more heavily on Black and Hispanic residents in Harris County, the most populous county in Texas. We found no consistent evidence or significant correlations between historic county-average PM_2.5_ concentration and COVID-19 incidence or death. Our findings suggest that public health and economic aid policy should consider the racially-segregated burden of disease to better mitigate costs and support equity for the duration and aftermath of health crises.

## Introduction

As of August 19, 2021, over 37 million people in the United States have been infected and more than 625,000 have died from COVID-19^1^. The resulting economic, health, and social costs are significant and long-lasting. These include the direct costs from lost life, the economic costs from the economic slowdown, health costs from missed treatments for other diseases, mental health issues associated with the lockdowns, and many other indirect cost. As we seek to recover and learn how to manage future pandemics, it is critical to document these costs at the national and international levels^2–6^.

Emerging evidence suggests that the effects from COVID-19 are not experienced equally among different segments of the US population, with low-income communities and communities of color experiencing a disproportionate burden from the disease^7–9^. Preliminary numbers from the American Public Media Research Lab, for example, suggest that the COVID-19 mortality rate for Black Americans is 2.3 times as high as the rate for white Americans^7^. These disparities may be the result of many different factors. First, the proximity, higher population density, and more frequent use of public transportation in many of the areas populated by minority communities can make disease transmission more likely^10–13^. A higher representation of people of color in essential jobs may also lead to further exposure^10–13^. Additional relevant variables may include differential access to healthcare and education, differences in financial status, and exposure to chronic stress^14^, including stress imposed by racial discrimination^15^. Finally, pre-existing conditions, which can develop from living in areas of high water and air pollution^16^ and unequal access to high quality healthcare, may lead to more serious outcomes for those who develop symptoms of COVID-19.

As mentioned above, some existing studies have investigated the correlation between COVID-19 incidents and several other factors. These include, for example, the use of public transport, race/ethnic minority status, housing prices, mean temperature, smoking status, age, access to healthcare, pollution, etc. Our approach is decidedly more focused. First, we restrict our analysis to the state of Texas. Second, we limit our analysis to two independent variables: race/ethnicity and local pollution (as measured by PM2.5 concentrations). Due to the focus of the local Houston economy on the energy sector, most of which is fossil fuel-related, and the emphasis on private transportation, Texas is a natural state to investigate the pandemic effects of local pollution. In addition, there is evidence that minorities and low income families are disproportionately located in areas that expose them to higher levels of pollution. However, our analysis does not rely on an a priori statistical relationship between race/ethnicity and pollution. Here we consider the effects of exposure to atmospheric particles with diameter smaller than 2.5 µm (PM_2.5_), a form of pollution associated with negative respiratory and cardiovascular outcomes^17^. Regional disparities in PM_2.5_ exposure remain, despite overall improvements in air quality over the past few decades^18^. Communities of color tend to have a higher percentage population in more heavily polluted regions^19^. Taken together, these factors raise concerns about health and environmental justice.

A fast-growing body of literature has documented the connections between COVID-19 and race/ethnicity. A recent US National Bureau of Economics Research study^8^ conducted a thorough, county-level analysis of possible socioeconomic contributors to COVID discrepancies. They found that education and occupation account for much of the COVID-19 variance that otherwise appears to be linked to variation in county-level percentage Hispanic and percentage Asian American population. However, differences in percentage Black and Native American/Alaskan population and the associated difference in COVID-19 outcomes were found to not be completely explained by socioeconomic factors. Examining a group served by a single hospital system^9^, one study found that an individual’s race/ethnicity (specifically, whether they are Black), together with "increasing age, a higher score on the Charlson Comorbidity Index (indicating a greater burden of illness), public insurance (Medicare or Medicaid), residence in a low-income area, and obesity" were positively correlated with higher rates of COVID-19 hospitalization. Finally, decades of research indicate that communities with higher populations of racial minorities historically have experienced higher burdens from a large range of disasters^20^. These burdens included higher morbidity and less successful recoveries from natural disasters^20^. We hypothesize that these patterns extend to the COVID-19 crisis.

The connection between COVID-19 and environmental racism is an important topic that deserves much further research, for example as has been performed by Knittel and Ozaltun^10^. These authors consider a large number of explanatory factors for the variance in COVID-19 death and case rates among different communities, including factors like race/ethnic minority and pollution. Our analysis begins to address some of the issues raised, but is more focused in a number of dimensions. First, as we concentrate on the state of Texas, we do not have to consider fixed effects and heterogeneity across states. Second, we only consider two independent variables: race/ethnicity and PM2.5 concentrations. Our statistical analysis allows for the results about race/ethnicity and pollution to be considered in isolation.

The majority of existing studies do not consider the economic costs from COVID-19 related deaths^21,22^. Addressing this, here we used the Value of Statistical Life (VSL) approach to quantify mortality-related costs from COVID-19. The VSL approach is increasingly used in insurance, as well as in cost–benefit analysis. It can inform public policy decisions by quantifying the costs resulting from deaths, or the benefits resulting from avoided deaths, say, as a result of employing costly mitigation measures. Our findings suggest that ethnic and racial minorities in Texas are suffering a disproportionate fraction of the economic costs associated with COVID-19-related mortality. While we will document these disproportionate costs, our analysis is silent as to why these are concentrated across minority and other vulnerable communities. This likely depends on a number of factors and calls for additional research on how systemic racism affects pandemic response and on how policy can mitigate such effects in the future. Combining these risk factors, this work evaluates the relationship between regional historic pollution levels, racial/ethnic composition, and COVID-19 incidence in the state of Texas. We investigate whether pollution and race/ethnicity are correlated with the observed variation in per capita COVID-19 incidence and deaths among the 254 counties in Texas. Our goal is not to explain all variation; many additional variables, such as proportion of residents considered essential workers, population density, healthcare access, and use of public transport likely influence COVID-19 incidence (see Knittel and Ozaltun^10^). Rather, we aim to examine whether two factors—race/ethnicity and PM_2.5_ levels—correlate with COVID-19 cases and deaths. Finally, we quantify the estimated economic costs of COVID-19, breaking down the cost experienced by the most populous county in Texas (Harris County) by race and ethnicity.

## Methods

### Data

The variables analyzed in this study include long-term particulate matter PM_2.5_ concentration, racial composition, and COVID-19-related incidents and deaths. The data were collected at the county level for all counties in Texas.

#### PM_2.5_

We used 2000–2016 average yearly PM2.5 level per county, in μg/m^3^, from the combined satellite, modeled, and monitored PM2.5 data model produced by Van Donkelaar et al.^23^ and used by Wu et al. in their COVID-19 study^21^. This data was downloaded from the Wu et al.^21^ Github (https://github.com/wxwx1993/PM_COVID-19), using the csv (comma-separated values) reader Python library. Data was then averaged across 2000–2016 for each county.

#### Race/ethnicity

We sourced county-level demographic data, which we defined as the percent of the county population that identify as Black and/or Hispanic, from 2019 US Census estimates of race and ethnicity by county^24^ (Supplementary Information). We studied those minority groups whose representation in the population allowed for a large enough disease incidence to conduct meaningful statistical analysis. We thus excluded Native American/Native Alaskan, as they constitute less than 1% of the state’s population^25^. Note that though Asian Americans constitute a larger portion of the Texas population (about 5%) and are a minoritized group, we also excluded this group in our regression model in order for our results to be directly comparable to existing related studies^10,25^. Total population values used to calculate racial-ethnic composition were sourced from the US Census dataset, while total population values for COVID-19 data were sourced from the Texas Department of State Health Services, TXDSHS^26^ (Supplementary Information). In addition to demography-based regressions, we calculated expected costs from COVID-19 deaths by racial/ethnic group for Harris County. The actual racial-ethnic breakdowns of COVID-19 deaths were sourced from Harris County's COVID-19 dashboard^27^; the estimated breakdowns, used for comparison, were generated from 2018 Census data points on racial/ethnic numbers broken down by age, also accessed on the US Census Bureau’s public data portal^28^.

#### COVID-19

We downloaded statewide COVID-19 data from the TXDSHS COVID-19 dashboard^26^, which formed the case and death data used in our regressions. Since numbers were updated daily, unlike the PM_2.5_ and demographic data, we also implemented Python’s Selenium library-based download process to clean and save the data. The final values of COVID-19 deaths used in this analysis were downloaded on July 26, 2020, and values of cases were downloaded on July 27, 2020. In our central analysis, we investigated the 50 most populous Texas counties (there are a total of 254 counties in Texas). The analysis is supplemented by robustness checks of the 30 most populous counties, as well as the 30 and 50 most polluted counties. These choices reflect the assumption that each of these groups consist of counties that are fairly comparable to each other in terms of both race/ethnicity, as well as pollution exposure. When considering the abundant factors that can influence COVID-19 outcomes, the counties within these groups likely share similar characteristics. We are mindful that by excluding other counties, we are missing critical impacts of structural racism and environmental injustice on racial/ethnic disparities in COVID-19 outcomes in these locations. In all, every county in our core analysis reported at least 1 COVID-19 case and death. To estimate the age- and racial-ethnic distributions of economic loss resulting from COVID-19 deaths in Texas and in Harris County, we collected age data from TXDSHS and racial-ethnic data from the Harris County COVID-19 dashboard^27^.

### Analysis

We employed multivariable, ordinary least squares (OLS) linear regression modeling using *R* to study the connection between race/ethnic minority status, local pollution, and county COVID-19-related cases and deaths. Our initial hypothesis is that race/ethnicity, as measured by the percentage of the local population that is Black/Hispanic, and pollution, as measured by higher long-term PM2.5 local concentrations, positively correlate with a higher incidence of COVID-19 deaths. Our hypothesis assumes a causal relationship between these variables. However, our regression analysis techniques formally permit us to interpret these relationships as correlations only. As mentioned above, assigning causal relationships between these variables would require a deep analysis of structural racism and its related underlying causes, analyses that are beyond the scope of the work presented here.

We examined both cases and related fatalities as dependent variables, and average PM_2.5_ levels and racial minority composition, which we defined as percentage of population Black and/or Hispanic, as independent variables. To mitigate COVID-19 variation that stems from population density, population mixing rates, and other urban–rural differences, our first regression includes only the top 50 most populous counties in Texas. Since we also wanted to mitigate variance in pollution levels, our supplementary regressions (see “Supplementary Files”, Sect. 2) include the top 50 most polluted counties in Texas based on 2000–2016 average PM_2.5_ levels. To check the sensitivity of our analysis to the number of counties employed, we also ran regressions with the top 30 most populous and top 30 most polluted counties (see “Supplementary Files”, Sect. 2).

To investigate the mortality-related economic costs of COVID-19, we used the Aldy and Viscusi^29^ method of age-discriminating values of statistical life (“Supplementary Table S1”). Dobson et al. use a similar methodology to calculate the global costs of the COVID-19 pandemic^2^. We then estimated the amount of value lost from COVID-19 deaths in Texas, given age-specific death breakdowns. Unfortunately, at the time of analysis, the state of Texas did not report complete race/ethnic minority information data in connection to COVID-19 deaths. However, data with such specificity was available for Harris County, the most populous county in Texas. We were then able to compute the estimated economic value lost by death by race/ethnicity, given age- and race/ethnic minority -specific COVID-19 death breakdowns. We removed deaths with unknown demographics (a negligible percentage of deaths for Texas as a whole and about 4% for Harris County, as of July 28, 2020) before accounting for total costs and percentage of costs.

## Results

### Race, pollution, and COVID-19 infection

Estimated regression coefficients for each of our independent variables—race/ethnic minority /ethnicity and average PM_2.5_, calculated using data from the top 50 most populous counties—are given in Tables 1 and 2. Regression coefficients for the most polluted counties are given in Supplementary Sect. 2 (Tables S2–Table S4). The Race variable represents the combined percent non-Hispanic Black with the percent Hispanic out of the total population of each county.

**Table 1:** Regression of PM_2.5,_ race, and COVID-19 deaths for the 50 most populous counties in Texas.

Multivariate linear regression of PM2.5, race/ethnicity, and COVID deaths for the 50 most *Populous* counties in Texas Dependent variable: COVID deaths Independent variables: PM2.5 (2000–2016 average in μg/m^3^), percent minority race/ethnicity (%Non-Hispanic Black + %Hispanic)				
Residuals				
Min	1Q	Median	3Q	Max
− 12.334	− 6.193	− 2.246	1.107	37.842

Coefficients	Estimate	Std. error	t-value	Pr( >|t|)
(Intercept)	5.36085	10.99702	0.487	0.628183
PM2.5	0.07970	1.22429	0.065	0.948373
Race/ethnicity	0.27962	0.07864	3.556	0.000872***

Signif. codes: 0 ‘***’ 0.001 ‘**’ 0.01 ‘*’ 0.05 ‘.’ 0.1 ‘ ’ 1.

Multiple R-squared: 0.2167, Adjusted R-squared: 0.1833.

Residual standard error: 10.35 on 47 degrees of freedom.

F-statistic: 6.5 on 2 and 47 degrees of freedom, p-value: 0.003219.

**Table 2:** Regression of PM_2.5_, race, and COVID-19 cases for the 50 most populous counties in Texas.

Multivariate linear regression of PM2.5, race/ethnicity, and COVID cases for the 50 most *Populous* counties in Texas Dependent variable: COVID cases Independent variables: PM2.5 (2000–2016 average in μg/m^3^), percent minority race/ethnicity (%Non-Hispanic Black + %Hispanic)				
Residuals				
Min	1Q	Median	3Q	Max
− 765.2	− 330.0	− 186.0	180.7	2448.2

Coefficients	Estimate	Std. error	t-value	Pr( >|t|)
(Intercept)	918.308	627.219	1.464	0.14982
PM2.5	− 36.997	69.828	− 0.530	0.59872
Race/ethnicity	16.022	4.485	3.572	0.00083***

Signif. codes: 0 ‘***’ 0.001 ‘**’ 0.01 ‘*’ 0.05 ‘.’ 0.1 ‘ ’ 1.

Multiple R-squared: 0.2135, Adjusted R-squared: 0.1801.

Residual standard error: 590.3 on 47 degrees of freedom.

F-statistic: 6.38 on 2 and 47 degrees of freedom, p-value: 0.003536.

**Figure 1:**
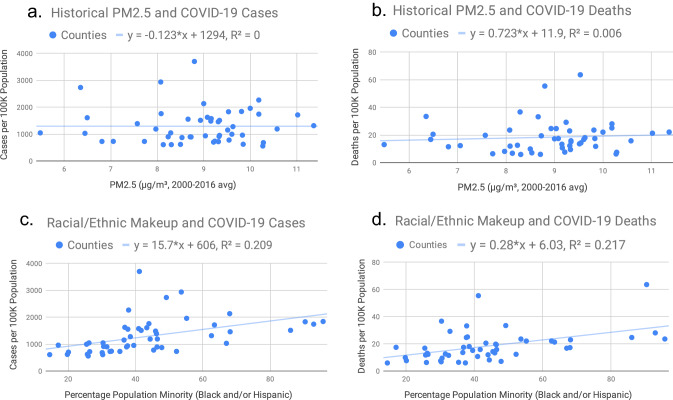
(Fig. 1**a**–1**d**) Correlation between race/ethnic minority, PM_2.5_, and COVID-19 for the 50 Most Populous Counties in Texas. (**a**) Historical PM2.5 and COVID-19 cases. (**b**) Historical PM2.5 and COVID-19 deaths. (**c**) Racial/ethnic makeup and COVID-19 cases. (**d**) racial/ethnic makeup and COVID-19 deaths.

For all regression analyses conducted, a statistically significant positive correlation emerges between race/ethnicity and COVID-19. With a coefficient of 0.28, the linear regression of Texas's 50 most populous counties suggests that for every 1% increase in a county's minority representation, counties see a 0.28 increase in death count per 100,000 people. As much as 18.33% of the variation in COVID-19 death rates among counties can be attributed to racial/ethnic makeup. The data also show a significant positive correlation between COVID-19 cases and race/ethnicity: for every 1% increase in minority population, counties saw a 16.02 increase in cases per 100,000 people. In parallel with COVID-19 deaths, 18.01% of the variation in case counts amongst populous Texas counties can be attributed to differences in racial/ethnic makeup. In contrast, we found no statistically significant correlation between long-term PM_2.5_ exposure and COVID-19. Analysis of fatalities in the same 50 counties produces a p-value of 0.9484, and data on cases produces a p-value of 0.5987. Figure 1 (1.1–1.4) provide scatterplots of the data used in the regression. Additionally, Figs. S1–Fig. S4 in the “Supplementary Information” render these relationships in a visual context, using heat map symbology to illustrate data for the two largest metropolitan areas in Texas.

### Economic costs due to COVID-19 in Texas

Next, we discuss estimates of the economic costs resulting from COVID-19 deaths, disaggregated by age and race/ethnic minority, based on Value-of-Statistical-Life (VSL) calculations. These figures exclusively capture COVID-19-related mortality costs and do not include other costs, for example, related to pollution, hospitalizations, lost income for reasons related to the pandemic, etc. There is a large literature in economics and actuarial science that makes use of VSL methodologies. Aldy and Viscusi^10^ review the market-based literature surrounding economic valuation of life, including an occupational risk-based model, as well as the US Environmental Protection Agency’s model that does not adjust by age. Aldy and Viscusi’s analysis^29^ (see “Supplementary Files”, Table S1), characterizing the value of life based on age, is based on a person or group’s willingness to pay for risk reduction. The resulting values tend to be lower than those used in studies that do not discount by age. Thus, we assert our numbers are conservative and should be viewed as a lower bound on the corresponding costs.

The age-based breakdown of costs from COVID-19 deaths (Table 3) provides a consistent picture of inequality associated with COVID-19 economic burden. First, if we consider age composition without taking race into account, we find that those aged 65 or older composed most of the cost—about 55.4% of the total $12.2 billion. The 45–54 age group comes in second, at 17.5% of the total, followed by ages 55–64, at 13.3%. The two groups contributing the least were the 1–4 years and < 1 year, at 0.00850% and 0.0341%, respectively.

**Table 3:** Age-specific economic costs of COVID-19 deaths for the state of Texas.

Age groupings	Number of deaths	% of total deaths	Cost from death (millions of dollars)	% of Texas total cost (%)
Under 1 year	2	0.03500787677	7.48	0.03
1–4 years	0.5	0.008751969193	1.87	0.01
5–14 years	4.5	0.07876772274	16.83	0.08
15–24 years	27.5	0.4813583056	102.85	0.47
25–34 years	84	1.470330824	792.12	3.61
35–44 years	218	3.815858568	2105.88	9.60
45–54 years	474.5	8.305618764	3829.22	17.46
55–64 years	850	14.87834763	2915.50	13.30
65 + years	4052	70.92595834	12,156.00	55.44
Total	5713	100	21,927.75	100.00

0.5 deaths exist here because of artificial age group delineations. Texas reports deaths in 10-year age groups, i.e. 1–9 years, 10–19 years, 20–29 years, etc. The age grouping we use, however, derives from Aldy and Viscusi^29^ (see Table S1 in “Supplemental Files”). The misalignment necessitates splitting of several age groups in two, creating the 0.5 deaths. It is common practice in VSL calculations to include all individuals, regardless of age. This is due to the nature of the VSL concept, which is meant to capture the value the society assigns to human life, instead of the resulting loss in life-time income/earnings.

Table 4 reports the racial burden of costs from COVID-19 deaths. Of the total economic cost in Harris County, the share of Black, Hispanic/Latinx, and Asian American/Pacific Islanders was higher than expected by their age-specific population share in Harris County. Black residents experienced 170.37% of the expected cost (a nominal 24.58% of the total cost compared to 14.43% of the expected total cost), Hispanic/Latinx experienced 138.27% of the expected cost (a nominal 42.34% instead of 32.79%), and Asian American/Pacific Islander experienced 100.84% of the expected cost (a nominal 5.59% instead of the expected 5.54% of total cost). By contrast, multiracial groups experienced the lowest percentage of expected cost given their population share and age makeup, at 13.23% of the expected cost (a nominal 0.22% of the total cost compared to the expected 1.69%), followed by White, at 53.20% of their expected cost (a nominal 24.05% compared to the expected 45.21%), and finally American Indian/Alaska Native, at 63.04% of the expected cost (a nominal 0.21% compared to the expected 0.34%). The cost for the Hispanic/Latinx and the Black populations are particularly stark. These findings suggest that the Hispanic/Latinx and Black communities in Harris County carry a disproportionate amount of the economic costs associated with COVID-19 fatalities. The lack of availability of statewide data on the racial composition of COVID-19 deaths prevented us from calculating the resulting economic costs for the entire state of Texas. However, we assert that the data clearly documents asymmetric distribution of these costs in Harris County. Whether this is also indicative for data in the entire state of Texas remains an open question given the differential racial/ethnic breakup of Texas, especially in more rural counties. (This caveat is driven by the fact that, to the best of our knowledge, the state of Texas has neither tracked nor made public county-level race/ethnicity data in connection to COVID-19 related deaths, with "county-level" referring to deaths broken down by county and tagged by race/ethnicity).

**Table 4:** Racial breakdown of actual and expected economic costs of COVID-19 deaths in Harris County.

Harris County economic cost of COVID deaths (in millions of dollars)			
Race/ethnicity	Percent actual of expected cost (%)	Percent actual of total cost (%)	Percent expected of total cost (%)
American Indian/Alaska Native	63.04	0.21	0.34
Asian American/Pacific Islander	100.84	5.59	5.54
Black	170.37	24.58	14.43
Hispanic/Latinx	138.27	45.34	32.79
White	53.20	24.05	45.21
Multi-racial	13.23	0.22	1.69

“Percent Expected of Total Cost” assumes uniform deaths across races/ethnicities and age groups.

## Discussion and conclusions

This study employs county data from Texas to investigate the relationship between particulate matter concentrations, race/ethnicity, and COVID-19 incidents and deaths. Whether we look at the largest Texas counties by population or by pollution levels, our results are consistent. From our multivariate regressions, we found no statistically significant relationship between county-average PM2.5 concentrations and COVID-19 cases or deaths in Texas. We did find strong evidence that the racial composition of a county is significantly correlated with COVID-19 outcomes, with a higher fraction of minority population correlating with more disease incidence and related deaths in the county. This finding is consistent with other reports on the racial inequities of COVID-19. Based on our Value of Statistical Life analysis, racial minority overrepresentation in COVID-19 deaths also translates to an overrepresentation in the economic costs of COVID-19 deaths. These findings support the hypothesis that racial minorities are shouldering a disproportionate amount of the costs of COVID-19.

Black/Hispanic minority status is highly correlated with a higher incidence of COVID-19, and highly correlated with a higher probability of death from the disease. Of course, disease incidents and deaths depend on many factors and the goal of our study was not to consider all possible explanatory factors; rather, our focus was to provide a first-pass analysis surrounding the connection between race/ethnicity, long-term air pollution exposure, and COVID-19 incidents and related fatalities in Texas. While aggregate studies over the entire US population are informative, aggregation can also hide some state-related heterogeneity. Focusing on a particular state can provide complementary information to large-scale national studies. We did not find a statistically significant relationship between COVID-19 and historical pollution levels. The findings of previous studies on this question are mixed, and we believe that more studies or natural experiments, perhaps with a higher level of granularity than ours—for example, by ZIP Code—are needed in order to settle this question.

We note that there is an important and growing literature connecting public health to environmental justice issues. Even when decoupled from pollution effects, the strong positive correlation between the percentage of the population identified as Black or Hispanic and COVID-19 mortality rates is a striking fact that deserves to be studied in more detail in the future. One central innovation of our approach involved using the value of statistical life (VSL) notion in order to monetize the losses associated with COVID-19-related mortality. To our knowledge, ours is the first study to calculate these costs in connection to racial/ethnicity disparities during a pandemic. We found important differences across groups, asserting that Black and Hispanic communities experience a disproportionately high economic burden during a pandemic. We emphasize that while indicative of the distribution of costs from COVID-19, the dollar figures should be considered a lower bound of the overall costs borne by communities of color. Even if VSL captured the entire cost from the loss of a human life, the effects on individuals and communities are likely to be long-lasting, especially since racial/ethnic minorities in the US are less likely to be wealthy^30^ compared to their white peers. Our results indicate that the economic costs of COVID-19 fall more heavily on these communities, suggesting that the fallout of COVID-19 exacerbates an existing, unequal racial distribution of wealth.

These implications can and must inform the development of public health policy related to the pandemic and future health threats. Considering ways to effectively direct resources towards communities with a higher minority representation can improve health equity, as well as minimize the total cost suffered from crises like pandemics. Although a cost–benefit analysis of such policies is beyond the scope of our paper, we can envision measures that would likely be beneficial in addressing existing disparities. These include equal access to regular healthcare, as well as specialized and emergency healthcare during pandemics and equal access to vaccinations, masks, and other protective equipment. The significant income inequality across racial/ethnic groups in the US almost certainly contributes to the problem. Our analysis is a resounding call for further research on ways to address racial disparities in public health.

While our PM_2.5_ results indicate that county-level average pollution is not correlated with COVID-19 severity, this conclusion could reflect a few broad assumptions. First, our analysis considers all COVID-19 deaths. However, the causes of COVID-19 related deaths can vary and can be due to respiratory or organ failure, immune response, cardiovascular, or other reasons. It is possible that local pollution is a contributing factor to some of these causes, but not others. Second, we assumed that every location within a county had the same long-term exposure to PM_2.5_ and the same experience of COVID-19. This assumption may not always hold for a variety of reasons. Upon examining two adjacent Harris County neighborhoods, Gulfton and Bellaire, there is a stark 6:1 ratio in COVID-19 death rate^31^. Studies by Brooks and Sethi^32^ and Chen and Krieger^33^ suggest that pollution levels and COVID-19 levels, respectively, vary across zip codes within the same county. Within-county variability is likely stronger in urban counties (in terms of both pollution and demographics), where there are local hotspots, than in rural counties where PM_2.5_ is mostly biogenic in nature. Furthermore, wealthier regions within a county may have better air quality than other areas populated with industries and factories which emit air toxics on a regular basis.

We acknowledge important caveats of this work. In any regression analysis there is always uncertainty driven by omitted variables, which can affect the consistency of OLS estimation. There are, of course, several other variables that may have an effect on COVID-19 cases and related deaths (for example, use of public transportation). However, for such explanatory variables to confound this analysis, they would need to also be highly correlated with local pollution or race/ethnic minority. Evaluation of these factors is beyond the scope of this work. Secondly, as mentioned above, this study employs PM_2.5_ spanning the period 2000–2016, which may not be accurately representative of the air pollution that people were exposed to over timescales relevant to their health during the pandemic. PM_2.5_ levels have sharply declined in areas influenced by power plants and roads, whereas wildfire PM_2.5_ has increased^34^. While our statewide averages are likely robust, spatial patterns are in fact at the core of this analysis, and gradients between counties have likely changed substantially, with urban regions likely getting cleaner far faster than rural ones^34^. We did not incorporate these details in the analysis presented here; in future work, this issue could be addressed by limiting the regression analysis to a more restricted time period spanning only more recent years where the explicit controls on spatiotemporal trends in PM_2.5_ are better constrained.

Furthermore, a more nuanced treatment of race and ethnicity is sorely needed in this and other forthcoming work. In particular, race/ethnicity classifications often identify individuals as a single race. More accurate classifications must allow for multi-racial classifications (e.g. both Black and Hispanic, Asian American). Our analysis does not consider individuals identifying as both Black/Hispanic, and the impacts of this shortcoming is difficult to quantify. Fixing this problem is contingent on local, state, and federal agencies appropriately documenting mixed-race members of the population. Mis-classification is likely driven by a lack of ability for individuals to self-identify as members of multiple races/ethnic groups.

Finally, we hope to study these questions at the zip-code level or other more granular resolution. This extension will likely shed greater light on the relationship between air pollution and COVID-19. Geographical resolution may also be relevant to other aspects of this study. For example, racial-ethnic correlations might be better described at higher resolution, given that researchers have noted that economic and demographic metrics in the US tend to vary greatly by zip code^8^. Finally, this study includes only two of many possible explanatory variables. Factors like COVID-19 policy and urbanization were not accounted for. To minimize some of this variance between counties, future studies should consider independent regressions for individual metropolitan areas. In view of recent developments, our analysis also points to the need for future research to study related effects resulting from COVID variants and vaccination trends. The state of Texas has also adopted less restrictive policies regarding mandating masks and social distancing compared to other US states. It would be interesting to study the correlation between race/ethnicity and COVID-19 deaths across states that followed different mitigation measures.

Our study confirms related racial discrepancies in the COVID-19 health and economic experiences across different segments of the Texas population. Although our statistical analysis is limited to identifying correlations, it is natural to question the underlying causes of these stark inequalities. Our results point to the importance of structural or systemic racism^35^. While structural racism in America has been a focus of extensive study, the COVID-19 experience identifies an additional focus related to how race/ethnic minority and the existing structure of US institutions determine health and economic outcomes during pandemics. As this is not the last pandemic we will experience, this is an important topic for future study. Clearly, this focus is beyond the scope of this paper.

In conclusion, the COVID-19 pandemic has provided a unique, though tragic, opportunity to study the indirect impacts, or by-products, of the myriad economic and environmental impacts that have accompanied quarantine and shelter-in-place orders. The current pandemic provided a crucial but short-lived research window through which we evaluated the short-term impacts of rapid environmental mitigation, and how environmental pollution and economic activity co-vary. The economic shut down provides a glimpse of what Earth’s environment and our climate system might look like in an aggressive, fast-paced carbon mitigation world. It also provides an opportunity to assess which sectors of our economy (e.g., energy production, the restaurant industry, or grocery supply chains) contribute maximally to environmental pollution, given explicit knowledge of closure and shelter-in-place policy timelines. We hope that the analyses presented here initiates similar studies in other states, such that the codependency of health, environment, and race/ethnic minority status may be further documented and ultimately informative to policymakers.

## Supplementary Information

Supplementary Information.
