# Supplementary material for: Race and ethnic minority, local pollution, and COVID-19 deaths in Texas
Source: Sci Rep. 2022 Jan 19;12:1002. doi: 10.1038/s41598-021-04507-x (PMC8770513; doi:10.1038/s41598-021-04507-x)
Supplement: Supplementary file 1 — Supplementary Information. [file 41598_2021_4507_MOESM1_ESM.docx]

**SUPPLEMENTARY INFORMATION**

**TITLE:** Race & Ethnic Minority, Local Pollution, and COVID-19 Deaths in Texas

**AUTHORS:** Annie Xu^1^, Chima Adiole^1^, Nathan Botton^2^, Ted Loch –Temzelides^3^, Sylvia G. Dee^1^, Caroline A. Masiello^1^, Mitchell Osborn^1^, Jiaqi Lu^1^, Joanne Zhou^1^, Samantha Breaux^1^, Mark A. Torres^1^, Daniel Cohan^4^

Affiliations:

1. Department of Earth, Environmental and Planetary Sciences, Rice University
2. Princeton University, Department of Operations Research and Financial Engineering
3. Department of Economics, Rice University
4. Department of Civil & Environmental Engineering, Rice University

**S1. DATA AVAILABILITY**

All data used in this paper was publicly available as of March 19, 2021.

**COVID-19 Data**

- Texas Statewide -
  <https://dshs.texas.gov/coronavirus/AdditionalData.aspx%C2%A0>
  - Cases and deaths available by age group or by county (breakdown of both parameters, separately)
  - Per capita values calculated from total population values listed here
- Harris County, updated link to slightly modified data - <https://covid-harriscounty.hub.arcgis.com/datasets/40256f837cd344d787b0414255b4f529/explore>
- Harris County, original link to data source- <https://harriscounty.maps.arcgis.com/apps/opsdashboard/index.html#/c0de71f8ea484b85bb5efcb7c07c6914>
  - Cases and deaths available by age group *and* by race/ethnicity for Harris County (breakdown of both parameters, together)
  - Note: this link, from which we sourced our data, has been archived. The Harris County / City of Houston COVID-19 Data Hub evolved over the course of 2020, such that data on the racial/ethnic and age breakdown of COVID cases and deaths is most likely still available on the first, updated link, but is dispersed throughout the updated data dashboard.

**PM2.5 Data**

Accessed from Wu et al. 2020's Github, <https://github.com/wxwx1993/PM_COVID>. File name: "county_pm2.5.csv". Original source <https://sites.wustl.edu/acag/datasets/surface-pm2-5/>.

**Race/Ethnicity Data**

Race/Ethnicity by Texas County, 2019- <https://www.census.gov/data/tables/time-series/demo/popest/2010s-counties-detail.html>. As indicated in the Census Bureau’s file format guide, the 2019 estimates for each county correspond to the age group denoted as “0” and the year denoted as “12”. We used the Census data's Tot_Pop column to derive percentages for racial/ethnic composition.

- To identify 2019 estimates, view age group "0" and year "12" for each county, and combine female and male counts.
- Tot_Pop column was used to derive percentage-composition. Note that total population values here differ from total population points sourced from COVID-19 data and used to calculate per capita values.

Race/Ethnicity and Age for Harris County, 2018 –

Hispanic/Latinx: <https://data.census.gov/cedsci/table?q=harris%20county%20hispanic%20AND%20age&tid=ACSDT1Y2019.B01001I&hidePreview=false>

Multiracial:

<https://data.census.gov/cedsci/table?q=harris%20county%20multi%20AND%20age&t=Two%20or%20More%20Races&tid=ACSDT1Y2019.B01001G&hidePreview=false>

Black alone: <https://data.census.gov/cedsci/table?q=harris%20county%20black%20AND%20age&tid=ACSDT1Y2019.B01001B&hidePreview=false>

Asian alone: <https://data.census.gov/cedsci/table?q=harris%20county%20asian%20AND%20age&tid=ACSDT1Y2019.B01001D&hidePreview=false>

White alone: <https://data.census.gov/cedsci/table?q=harris%20county%20white%20AND%20age&tid=ACSST1Y2019.S0101&hidePreview=false>

American Indian/Alaska Native alone: <https://data.census.gov/cedsci/table?q=harris%20county%20american%20indian%20AND%20age&tid=ACSDT1Y2019.B01001C&hidePreview=false>

Native Hawaiian and Other Pacific Islander alone: <https://data.census.gov/cedsci/table?q=harris%20county%20pacific%20islander%20AND%20age&tid=ACSDT5Y2019.B01001E&hidePreview=false>

**S2.TABLES AND FIGURES**

| **Value of Statistical Life by Age (Aldy and Viscusi 2007)** | |
| --- | --- |
| **Age** | **Value of Life (Millions of Dollars)** |
| Under 1 year | 3.74 |
| 1-4 years | 3.74 |
| 5-14 years | 3.74 |
| 15-24 years | 3.74 |
| 25-34 years | 9.43 |
| 35-44 years | 9.66 |
| 45-54 years | 8.07 |
| 55-64 years | 3.43 |
| 65+ years | 3.00 |

**Table S1**. Age-discriminating Value of Statistical Life (Aldy and Viscusi 2007)

**Note:** Aldy and Viscusi's age groupings differ from the reported COVID-19 age group breakdowns; in order to align COVID-19 data to Aldy and Viscusi's model, we assumed that deaths were distributed uniformly *within* each age group, and then split the necessary age groups in half. For example, if 20 deaths occurred for those aged 30-39 years old, we assumed that 10 occurred among those aged 30-34 and 10 among 35-39.

**Tables S2.1-S2.2: Regressions for the 50 Most Polluted Counties in Texas**

| **Multivariate Linear Regression of PM2.5, Race/Ethnicity, and COVID Deaths for the 50 Most *Polluted* Counties in Texas**  **Dependent variable**: COVID Deaths  **Independent variables:** PM2.5 (2000-2016 average in μg/m³), Percent Minority Race/Ethnicity (%Non-Hispanic Black + %Hispanic) | | | | |
| --- | --- | --- | --- | --- |
|  | | | | |
| **Residuals:** |  |  |  |  |
| *Min* | *1Q* | *Median* | *3Q* | *Max* |
| -26.658 | -5.916 | -0.548 | 6.184 | 38.223 |
| **Coefficients:** |  |  |  |  |
|  | *Estimate* | *Std. Error* | *t-value* | *Pr(>\|t\|)* |
| *(Intercept)* | 29.45710 | 25.74228 | 1.144 | 0.258290 |
| *PM2.5* | -2.53805 | 2.79253 | -0.909 | 0.368054 |
| *Race/Ethnicity* | 0.29827 | 0.07594 | 3.928 | 0.000279 *** |
|  |  |  |  |  |
| Signif. codes: 0 ‘***’ 0.001 ‘**’ 0.01 ‘*’ 0.05 ‘.’ 0.1 ‘ ’ 1 | | | | |
| **Multiple R-squared:** 0.2487, **Adjusted R-squared:** 0.2167 | | | | |
| **Residual standard error:** 13.11 on 47 degrees of freedom | | | | |
| **F-statistic:** 7.779 on 2 and 47 DF, **p-value:** 0.001207 | | | | |

**Table S2.1** Regression of PM2.5, Race, and COVID-19 Deaths for the 50 Most Polluted Counties in Texas

| **Multivariate Linear Regression of PM2.5, Race/Ethnicity, and COVID Cases for the 50 Most *Polluted* Counties in Texas**  **Dependent variable**: COVID Cases  **Independent variables:** PM2.5 (2000-2016 average in μg/m³), Percent Minority Race/Ethnicity (%Non-Hispanic Black + %Hispanic) | | | | |
| --- | --- | --- | --- | --- |
|  | | | | |
| **Residuals:** |  |  |  |  |
| *Min* | *1Q* | *Median* | *3Q* | *Max* |
| -1225.8 | -345.7 | -147.6 | 299.5 | 2448.2 |
| **Coefficients:** |  |  |  |  |
|  | *Estimate* | *Std. Error* | *t-value* | *Pr(>\|t\|)* |
| *(Intercept)* | 1341.958 | 1319.842 | 1.017 | 0.3145 |
| *PM2.5* | -81.313 | 143.177 | -0.568 | 0.5728 |
| *Race/Ethnicity* | 15.204 | 3.893 | 3.905 | 0.0003 *** |
|  |  |  |  |  |
| Signif. codes: 0 ‘***’ 0.001 ‘**’ 0.01 ‘*’ 0.05 ‘.’ 0.1 ‘ ’ 1 | | | | |
| **Multiple R-squared:** 0.2511, **Adjusted R-squared:** 0.2192 | | | | |
| **Residual standard error:** 672.4 on 47 degrees of freedom | | | | |
| **F-statistic:** 7.879 on 2 and 47 DF, **p-value:** 0.00112 | | | | |

**Table S2.2**  Regression of PM2.5, Race, and COVID-19 Cases for the 50 Most Polluted Counties in Texas

**Tables S3.1-S3.2: Regressions for the 30 Most Polluted Counties in Texas**

| **Multivariate Linear Regression of PM2.5, Race/Ethnicity, and COVID Deaths for the 30 Most *Polluted* Counties in Texas**  **Dependent variable**: COVID Deaths  **Independent variables:** PM2.5 (2000-2016 average in μg/m³), Percent Minority Race/Ethnicity (%Non-Hispanic Black + %Hispanic) | | | | |
| --- | --- | --- | --- | --- |
|  | | | | |
| **Residuals:** |  |  |  |  |
| *Min* | *1Q* | *Median* | *3Q* | *Max* |
| -25.266 | -3.336 | -0.682 | 2.289 | 32.713 |
| **Coefficients:** |  |  |  |  |
|  | *Estimate* | *Std. Error* | *t-value* | *Pr(>\|t\|)* |
| *(Intercept)* | 13.47029 | 28.42046 | 0.474 | 0.63934 |
| *PM2.5* | -0.94327 | 2.97559 | -0.317 | 0.75368 |
| *Race/Ethnicity* | 0.29185 | 0.07941 | 3.675 | 0.00104 ** |
|  |  |  |  |  |
| Signif. codes: 0 ‘***’ 0.001 ‘**’ 0.01 ‘*’ 0.05 ‘.’ 0.1 ‘ ’ 1 | | | | |
| **Multiple R-squared:** 0.347, **Adjusted R-squared:** 0.2987 | | | | |
| **Residual standard error:** 10.68 on 27 degrees of freedom | | | | |
| **F-statistic:** 7.175 on 2 and 27 DF, **p-value:** 0.003169 | | | | |

**Table S3.1** Regression of PM2.5, Race, and COVID-19 Deaths for the 30 Most Polluted Counties in Texas

| **Multivariate Linear Regression of PM2.5, Race/Ethnicity, and COVID Cases for the 30 Most *Polluted* Counties in Texas**  **Dependent variable**: COVID Cases  **Independent variables:** PM2.5 (2000-2016 average in μg/m³), Percent Minority Race/Ethnicity (%Non-Hispanic Black + %Hispanic) | | | | |
| --- | --- | --- | --- | --- |
|  | | | | |
| **Residuals:** |  |  |  |  |
| *Min* | *1Q* | *Median* | *3Q* | *Max* |
| -1024.51 | -299.54 | -32.65 | 224.13 | 1145.61 |
| **Coefficients:** |  |  |  |  |
|  | *Estimate* | *Std. Error* | *t-value* | *Pr(>\|t\|)* |
| *(Intercept)* | 587.638 | 1305.083 | 0.450 | 0.65611 |
| *PM2.5* | 6.337 | 136.641 | 0.046 | 0.96335 |
| *Race/Ethnicity* | 12.321 | 3.647 | 3.379 | 0.00223 ** |
|  |  |  |  |  |
| Signif. codes: 0 ‘***’ 0.001 ‘**’ 0.01 ‘*’ 0.05 ‘.’ 0.1 ‘ ’ 1 | | | | |
| **Multiple R-squared:** 0.3226, **Adjusted R-squared:** 0.2724 | | | | |
| **Residual standard error:** 490.3 on 27 degrees of freedom | | | | |
| **F-statistic:** 6.429 on 2 and 27 DF, **p-value:** 0.005204 | | | | |

**Table S3.2** Regression of PM2.5, Race, and COVID-19 Cases for the 30 Most Polluted Counties in Texas

**Tables S4.1-S4.2: Regressions for the 30 Most Populated Counties in Texas**

| **Multivariate Linear Regression of PM2.5, Race/Ethnicity, and COVID Deaths for the 30 Most *Populous* Counties in Texas**  **Dependent variable**: COVID Deaths  **Independent variables:** PM2.5 (2000-2016 average in μg/m³), Percent Minority Race/Ethnicity (%Non-Hispanic Black + %Hispanic) | | | | |
| --- | --- | --- | --- | --- |
|  | | | | |
| **Residuals:** |  |  |  |  |
| *Min* | *1Q* | *Median* | *3Q* | *Max* |
| -10.134 | -4.817 | -2.250 | 1.772 | 32.650 |
| **Coefficients:** |  |  |  |  |
|  | *Estimate* | *Std. Error* | *t-value* | *Pr(>\|t\|)* |
| *(Intercept)* | -1.33685 | 14.18424 | -0.094 | 0.92561 |
| *PM2.5* | 0.42516 | 1.50697 | 0.282 | 0.78000 |
| *Race/Ethnicity* | 0.31206 | 0.08724 | 3.577 | 0.00134 ** |
|  |  |  |  |  |
| Signif. codes: 0 ‘***’ 0.001 ‘**’ 0.01 ‘*’ 0.05 ‘.’ 0.1 ‘ ’ 1 | | | | |
| **Multiple R-squared:** 0.3291, **Adjusted R-squared:** 0.2794 | | | | |
| **Residual standard error:** 9.38 on 27 degrees of freedom | | | | |
| **F-statistic:** 6.621 on 2 and 27 DF, **p-value:** 0.004574 | | | | |

**Table S4.1** Regression of PM2.5, Race, and COVID-19 Deaths for the 30 Most Populated Counties in Texas

| **Multivariate Linear Regression of PM2.5, Race/Ethnicity, and COVID Cases for the 30 Most *Populous* Counties in Texas**  **Dependent variable**: COVID Cases  **Independent variables:** PM2.5 (2000-2016 average in μg/m³), Percent Minority Race/Ethnicity (%Non-Hispanic Black + %Hispanic) | | | | |
| --- | --- | --- | --- | --- |
|  | | | | |
| **Residuals:** |  |  |  |  |
| *Min* | *1Q* | *Median* | *3Q* | *Max* |
| -533.1 | -310.8 | -105.1 | 266.8 | 1038.4 |
| **Coefficients:** |  |  |  |  |
|  | *Estimate* | *Std. Error* | *t-value* | *Pr(>\|t\|)* |
| *(Intercept)* | 228.705 | 625.773 | 0.365 | 0.71760 |
| *PM2.5* | 54.119 | 66.484 | 0.814 | 0.42275 |
| *Race/Ethnicity* | 11.782 | 3.849 | 3.061 | 0.00494 ** |
|  |  |  |  |  |
| Signif. codes: 0 ‘***’ 0.001 ‘**’ 0.01 ‘*’ 0.05 ‘.’ 0.1 ‘ ’ 1 | | | | |
| **Multiple R-squared:** 0.2836, **Adjusted R-squared:** 0.2305 | | | | |
| **Residual standard error:** 413.8 on 27 degrees of freedom | | | | |
| **F-statistic:** 5.343 on 2 and 27 DF, **p-value:** 0.0110 | | | | |

**Table S4.2** Regression of PM2.5, Race, and COVID-19 Cases for the 30 Most Populated Counties in Texas

In **Figures S1 – S4**, we focus on two of Texas's largest metropolitan areas -- the Dallas and Houston regions -- visualizing the correlation between race, pollution levels, and COVID-19 case and death incidents by county. Race and long-term PM2.5 averages are represented by coloration in red or green, respectively, and COVID-19 cases and deaths are represented by shaded circles.

Note: the scale of the case and death circles differ. For the sake of readability, when representing the same number of incidents, "death" markers are 10 times larger than "case" markers. For most counties, cases exceed deaths nearly 100-fold. The resulting figures show markers of readable size.



**Figure S1.** Historical PM2.5 overlaid with COVID-19 Deaths and Cases by County. Houston Metro Area. Figure generated using Plotly in Python (<https://plotly.com/python/>) and finalized using Software developed for this work: <https://rice-e-covid-tracker.herokuapp.com/>



**Figure S2.** Historical PM2.5 overlaid with COVID-19 Deaths and Cases by County. Dallas Metro Area. Figure generated using Plotly in Python (<https://plotly.com/python/>) and finalized using Software developed for this work: <https://rice-e-covid-tracker.herokuapp.com/>



**Figure S3.** Racial/Ethnic Composition overlaid with COVID-19 Deaths and Cases by County. Houston Metro Area. Figure generated using Plotly in Python (<https://plotly.com/python/>) and finalized using Software developed for this work: <https://rice-e-covid-tracker.herokuapp.com/>



**Figure S4.** Racial/Ethnic Composition overlaid with COVID-19 Deaths and Cases by County. Dallas Metro Area. Figure generated using Plotly in Python (<https://plotly.com/python/>) and finalized using Software developed for this work: <https://rice-e-covid-tracker.herokuapp.com/>

**
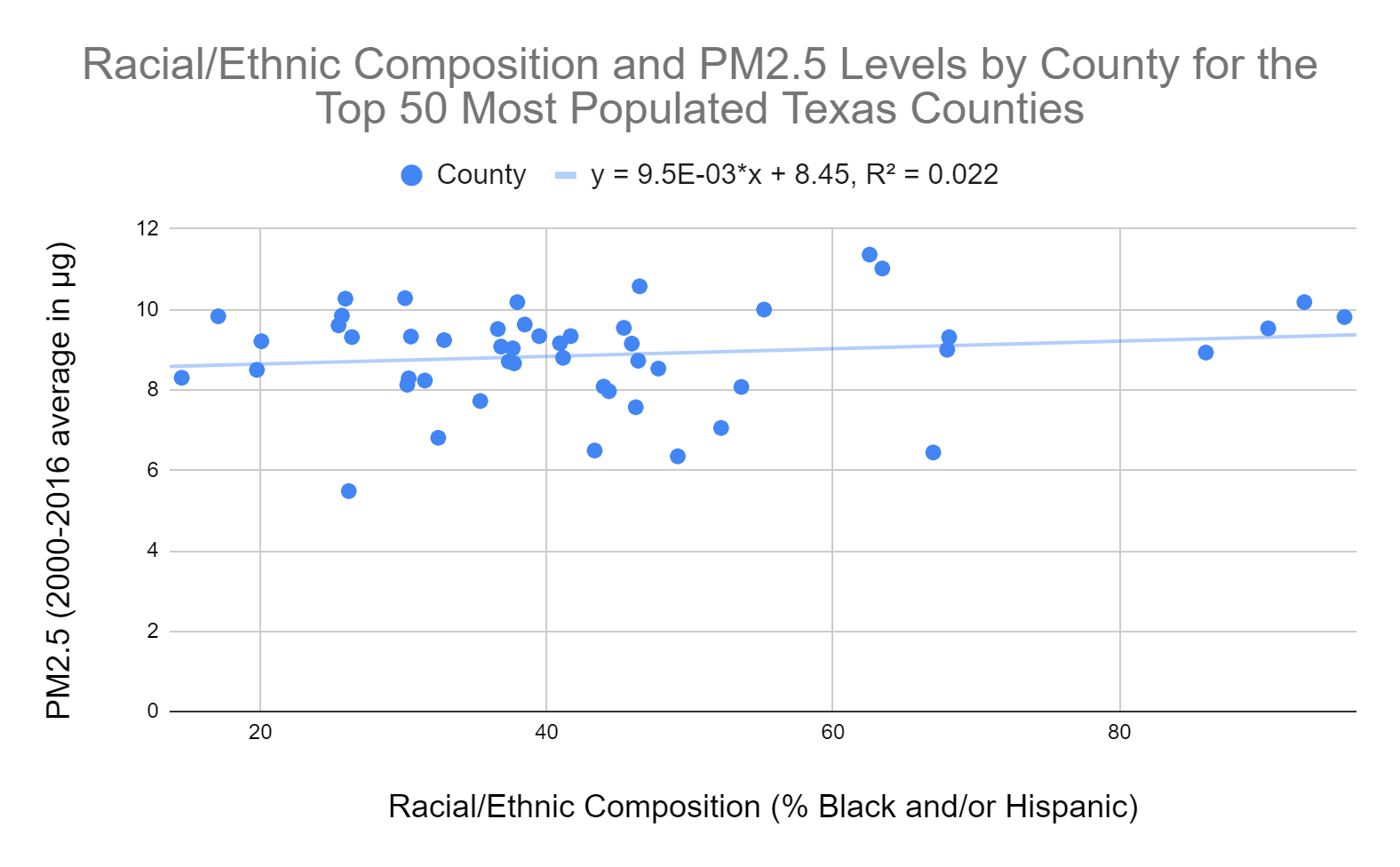
Figure S5.** Racial/Ethnic Composition plotted against PM2.5 Levels for the Top 50 Most Populated Texas Counties
